# Assessing Amino Acid Metabolism in Splanchnic Tissues and Mammary Glands to Short-Term Graded Removal of Lys From an Abomasal-Infused Amino Acid Mixture in Lactating Goats

**DOI:** 10.3389/fvets.2022.929587

**Published:** 2022-06-17

**Authors:** Yantao Li, Xueyan Lin, Chen Liu, Zhiyong Hu, Qiuling Hou, Zhonghua Wang

**Affiliations:** Ruminant Nutrition and Physiology Laboratory, College of Animal Science and Technology, Shandong Agricultural University, Taian, China

**Keywords:** lysine, lactating goats, amino acids, splanchnic tissues, mammary glands

## Abstract

To investigate the responses of amino acid metabolism in portal-drained viscera (PDV), liver, and mammary glands (MGs) to a graded gradual decrease of post-ruminal Lys supply, four multi-catheterized lactating goats were used in a 4 × 4 Latin square experiment. Goats were fasted for 12 h and then received a 33-h abomasal infusion of an amino acid mixture and glucose. Treatments consisted of a graded decrease of Lys content in the infusate to 100 (complete), 60, 30, or 0% as in casein. Lys-removed infusions decreased the production of milk, milk protein, fat, and lactose linearly and also decreased arterial Lys concentrations linearly (*p* < 0.05). Net PDV uptake decreased linearly (*p* < 0.05) with decreasing PDV loss ratio (*p* < 0.05). Although liver removal of Lys decreased linearly (*p* < 0.05), the removal ratio relative to portal absorption changed small, which was about 10% in all four treatments. Reduced Lys supply resulted in a linear decrease in the utilization of Lys in the peripheral tissues (except mammary, *p* < 0.05) and the release of more Lys in MGs. Although net mammary uptake of Lys declined linearly (*p* < 0.05), lactating goats can partially offset the negative effect of decreased circulating Lys concentrations by increasing mammary affinity (*p* < 0.05) and increasing mammary blood flow (*p* < 0.05). Graded removal of Lys from the infusate linearly decreased mammary uptake-to-output ratios of Lys (*p* < 0.05) suggesting that mammary catabolism of Lys decreased. Meanwhile, the treatments linearly increased circulating concentrations of glucagon and linearly decreased prolactin (*p* < 0.05). In conclusion, the results of the present study indicated that there were several mechanisms used to mitigate a Lys deficiency, including reduced catabolism of Lys in PDV and peripheral tissues (including MGs) and linearly increased mammary blood flow and mammary affinity together with increased mammary uptake and U:O of branched-chain amino acids (BCAA). Given these changes, the decline in milk protein production could be attributed to the combined effect of mass action with Lys and hormonal status.

## Introduction

Lactating ruminants usually have lower efficiency of nitrogen (N) utilization than growing animals, which result in a big environmental problem, such as soil and water eutrophication of N (1, 2). Postabsorptive efficiency of N use for milk is determined by the amount of metabolizable protein (MP) supplied relative to animal needs, the profile of absorbed EAA, and the rate of protein synthesis based on the present knowledge of amino acid (AA) nutrition (3), so improving the postabsorptive N efficiency without sacrificing milk protein production is a major strategy for increasing overall dietary N efficiency.

The losses of absorbed AA mainly occur in the portal-drained viscera (PDV), liver, peripheral tissues, and metabolic pathways other than only milk protein synthesis in the mammary glands (MGs) (4). In adult lactating cows, the net AA consumption from the PDV, liver, and MG was roughly equal to absorption, while the loss of AA metabolism in other tissues was low (5). Therefore, predicting changes in the rate and extent of AA metabolism by the PDV, liver, and MGs is an essential prerequisite for improving the precision of models that predict the MP requirements of dairy ruminants (6, 7).

Total AA losses during absorption are proportional to MP supply (4), but there is a variation in gut-associated losses among individual AA (8) that the metabolic disappearance rate of each EAA flowing through PDV ranges from 10.3 to 45.8% (7). Analysis of related research results shows that the liver catabolizes about 45% of the total AA flowing into the portal vein (4), but for individual EAA, the catabolized ratio varies widely, ranging from −0.4 to 55.2% (7). Changes in plasma AA profiles in response to casein infusion indicate that His, Met, and Phe plus Tyr are removed by the liver in substantial proportions of their net portal appearance, whereas Ile, Leu, Lys, and Val are barely extracted by the liver (9). Therefore, the relationship between individual EAA supply and metabolic loss in PDV and liver needs further investigation.

The mammary uptake to milk output ratio (U:O) of total amino acids is 96%−105%, indicating that the catabolism of amino acids does not occur under basically normal physiological conditions; the mammary uptake to milk output ratio of EAA and nonessential amino acids (NEAA) is 66%−79% and 141%−182%, respectively, indicating that part of EAA is converted to NEAA in the MGs (10). In general, Lys and branched-chain amino acids (BCAA) are taken up in excess relative to milk output, whereas Met, His, Phe, and Trp are taken up in approximately the same amount as in milk protein secretion (11, 12). Due to the EAA supply balance regulation mechanism of EAA supply at tissue and cell levels, in a certain range, the change of a single EAA supply may not lead to a change in milk protein production, but it will affect the lactational efficiency of the EAA.

Lys is often the limiting AA for milk protein synthesis in lactating ruminants. Increasing Lys in MP to more optimal concentrations by post-ruminal infusion (13, 14) or by feeding a rumen-protected Lys supplement (15, 16) increased milk protein yield.

Nitrogen originating from Lys appeared in Arg, Asp, Ala, Glu, and Ser concomitant with excess mammary uptake of Lys in dairy cows (17). As the deletion of Lys from an AA mixture decreased milk protein yield (18), this might suggest the excess uptake of Lys across the MGs may be obligate to maintain milk protein production by supporting NEAA synthesis.

Starvation-refeeding experiments, in which animals receive nutrients per os or by infusion after being deprived for 12–48 h, have been very useful in illustrating the postprandial response of dietary components (19, 20). It has been widely used in lactating rats, goats, and cows (21–24). The objective of this study was to determine how the PDV, liver, and MGs respond to varying short-term supplies of Lys in catheterized dairy goats. As Lys supply varies, splanchnic tissues and MGs are hypothesized to actively regulate AA catabolism.

## Materials and Methods

### Animals and Diet

Four multiparous Laoshan dairy goats averaging 60 ± 10 day in milk (DIM) and 50 ± 5 kg of body weight (BW) were used at the beginning of the experiment. Surgeries were performed at least 1 month before the start of the experiment in which all goats were surgically implanted with medical silicone catheters (1 mm × 2 mm, Chensheng Medical, China) in the portal vein, hepatic vein, and one mesenteric vein (25). A T-type Tygon (8 mm × 10 mm, Weili, China) was surgically implanted in the abomasum, and the right carotid artery was also raised to a subcutaneous position to provide access to arterial blood. The diet was formulated according to Agricultural and Food Research Council (AFRC) (26), which could provide 10.65 MJ ME and 110.7 g MP/kg DM (Table 1). The daily amount of diet offered was adjusted to maintain a 5% refusal rate. Goats had ad libitum access to water throughout the experiment.

**Table 1:** Ingredients, nutrients, and essential amino acid (AA) compositions of the pelleted diet.

**Ingredients**	**% of DM**	**Essential AA**	**g/kgDM**
Corn	14.00	Lys	8.10
Soybean meal	10.55	Met	2.51
Wheat bran	12.44	His	3.87
Alfalfa hay	28.94	Arg	9.57
Peanut vine	32.05	Thr	7.02
Salt	0.400	Val	9.04
4% Premix^1^	1.600	Ile	6.52
**Nutrients**	**Concentration**	Leu	12.21
ME, MJ/kg DM^2^	10.65	Phe	7.64
MP, g/kg DM^2^	110.7	Trp	2.14
CP, % of DM^3^	16.02	Lys, % of MP	7.32
NDF, % of DM^3^	33.83	Met, % of MP	2.27
ADF, % of DM^3^	24.33		

*^1^Premix contains (per kg of DM): 150 KIU VA, 250 KIU VD_3_, 2,400 mg VE, 2,000 mg nicotinic acid, 2,000 mg Fe, 2,500 mg Mn, 1,000 mg Cu, 3,600 mg Zn, 100 mg Se, 180 mg I, and 40 mg Co.*

*^2^Data were calculated according to NRC models (2001).*

*^3^Laboratory determined data*.

### Experimental Design and Procedure

**Figure 1:**
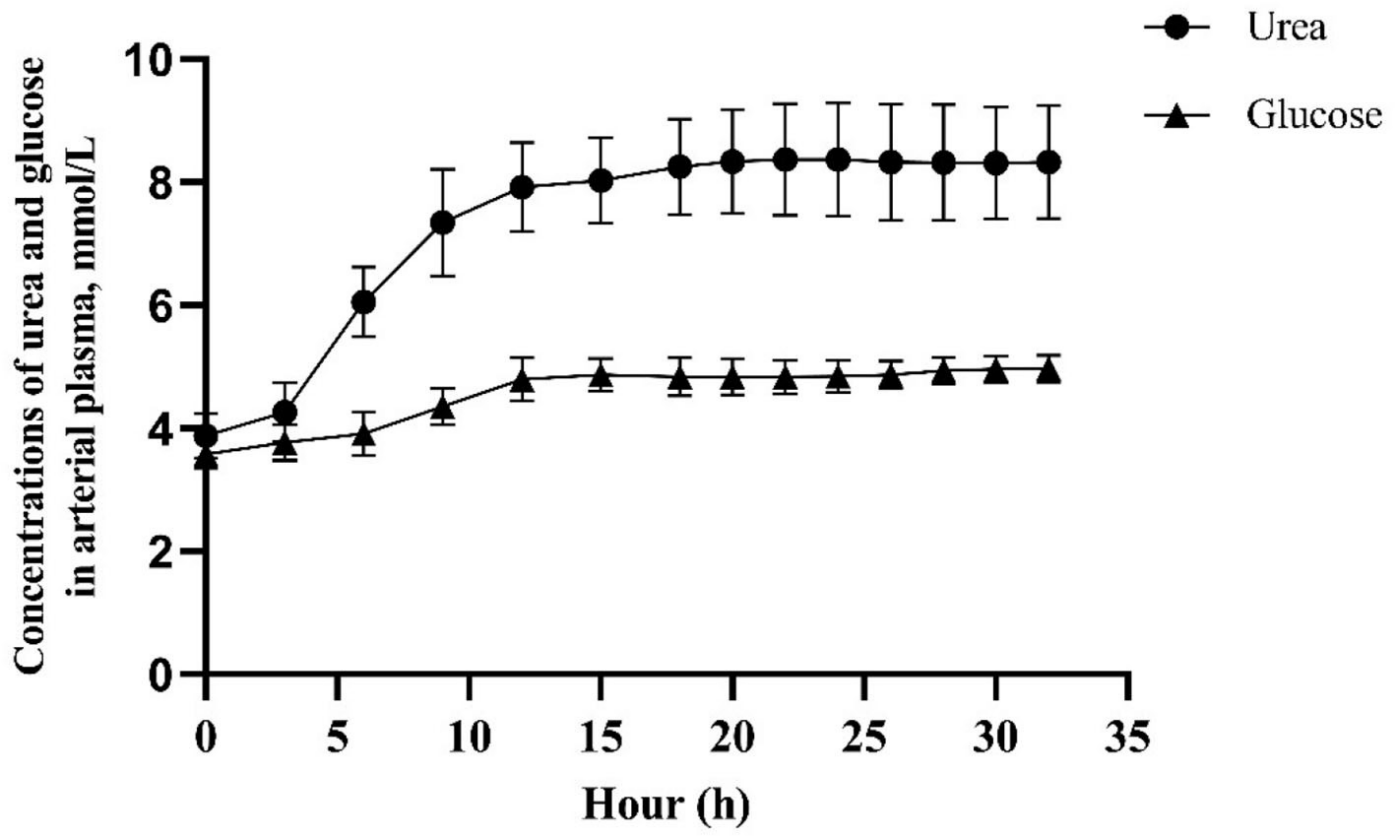
Concentrations of urea and glucose in arterial plasma after infusion of glucose and amino acid into abomasum in a preliminary experiment.

The experimental design was a randomized 4 × 4 Latin square with four 14-day periods. Treatments consisted of abomasal infusion of an AA mixture containing 100 (complete), 60, 30, or 0% of the Lys present in casein. The profile of casein was similar in composition to bovine milk casein in Table 2 (27). Lys was substituted by equal moles of Glu to achieve Lys-deficient infusates and, because of solubility limitations, all the Tyr was replaced by Phe. The infusion rate was set to match the MP requirement of each goat according to AFRC (26) based on milk yield recorded for 3 consecutive days before the start of infusion in each period. The amount of glucose infused was calculated to provide 3.6 mg glucose/kg BW per minute according to the observed metabolic use rate measured in lactating goats by using isotopic tracer technology (28). In each experimental period, goats were moved to individual metabolic cages and fasted for 12 h before the start of the infusion to mitigate possible interference from absorbed Lys from the remaining digesta. The infusion began at 08:00 on the 1st day and lasted 33 h to ensure steady blood glucose and urea concentration, which need about 15 h for glucose and 20 h for urea to establish according to the result of our pre-experiment (Figure 1). Samples were collected on the 2nd day. After stopping the infusion, goats were allowed for a 12-day rest in individual free stalls before the start of the next period.

**Table 2:** Amino acid profile of the complete amino acid mixture^1^.

**EAA**	**Content, g/100 g**	**NEAA**	**Content, g/100 g**
Lys	7.49	Ala	2.82
Met	2.82	Gly	1.78
Leu	8.89	Glu	9.95
Ile	4.61	Gln	9.80
Val	6.24	Asn	3.40
Thr	3.86	Asp	6.83
Phe	9.22	Ser	5.05
His	2.82	Pro	8.32
Arg	3.57	Cys	0.59
Trp	1.49		

*^1^Adapted from Bequette et al. (27)*.

The AA mixture and glucose were dissolved in 1,920 ml of saline each day, which was adjusted to a pH of 7.4 using HCl and NaOH and was infused continuously through a peristaltic pump (Type H-L2, Huxi Analytical Instrument Factory, Shanghai, China) to abomasum at a rate of 80 ml/h for the duration of the study. During the resting periods, goats were milked two times daily at 08:00 and 18:00 and allowed free access to the pelleted diet and water.

### Sampling and Analysis

Samples were collected on the 2nd day during each infusion period. Milk samples were collected between 08:00 and 17:00, which was weighed, recorded, and sampled for later analysis. Milk was divided into two subsamples: one was used for routine analysis using an infrared milk composition analyzer (Type 76110, FOSS, Denmark) and the other for AA determination after hydrolysis using an AA analyzer (Type L-8900, Hitch, Japan). Samples for AA analysis were sent to be hydrolyzed with hydrochloric acid at a final concentration of 6 M. The hydrolysate was filtrated through a 0.2-μm film before being loaded into the AA analyzer. To determine plasma flow across the splanchnic tissues, para-amino hippuric acid (pAH; 0.48g/h, 1% wt/vol) was continuously infused into the mesenteric vein catheter using a syringe pump (Type 50F6, SN, China) from 10:00 to 16:00 h. The pAH was infused 1.5 h before the first blood sample was collected, followed by a priming dose (0.2 g) for 5 min. On each sampling day, four sets of arterial, portal venous, and hepatic venous blood samples were collected simultaneously at 90-min intervals beginning at 11:30. Mammary venous blood samples were taken by venipuncture immediately after the withdrawal of the splanchnic blood samples. Blood samples were also divided into two samples, one was placed on ice and centrifuged (10 min, 1,800 × g at 4°C) within 30 min of collection to yield plasma, and the other one was for the packed cell volume by the automatic analyzer of blood cell (Mindray, BC-6100Plus). A total of four samples were pooled together and kept at −80°C for later analysis of free AA concentration using an AA analyzer (Type L-8900, Hitch, Japan). Samples were deproteinized by mixing with equal amounts of 10% sulfosalicylic acid, kept at 4°C overnight, and centrifuged at 15,000 × g for 20 min. The extracted supernatant was passed through a 0.2-μm film before being loaded into the AA analyzer. Arterial plasma was subsampled for total protein, albumin, urea-N, and glucose analysis by an automatic biochemical analyzer (Type 7020, Hitch, Japan). Hormones such as insulin, glucagon, and prolactin were determined by radioimmunoassay using commercial assay kits (Tianjin Xiehe Medical Technology Co. Ltd.).

### Calculations and Statistical Procedures

Blood and plasma flow were calculated from the downstream dilution of pAH infused into mesenteric catheters (29). Daily averages of blood flows were used to calculate the net fluxes of AA. Mammary plasma flow was estimated according to the Fick principle using Phe and Tyr as internal markers (30). The net fluxes of AA across the PDV, liver, total splanchnic tissues (TSP), and MG were calculated for each goat period as the product of the average plasma venous–arterial concentration difference and the average blood flow. A negative flux indicates utilization or removal, whereas a positive flux indicates net production or release of the nutrient across the tissue (31). Mammary uptake to milk output ratios (U:O) for individual AA were calculated as the mammary uptake of AA divided by the amount secreted in milk during the 9 h between milkings. PDV clearance rates (*K*_p_), liver clearance rates (*K*_h_), and mammary clearance rates (*K*_m_) were calculated to assess tissue affinity for blood metabolites (32):


$$\begin{array}{l} \;{\text{K}}_{\text{p}},\text{ L/h = }[\text{Abs +}\left({\text{C}}_{\text{a}}{\text{- C}}_{\text{p}}\right){\text{F}}_{\text{p}}]\;{\text{/C}}_{\text{p}}\\ {\text{K}}_{\text{h}},\text{ L/h = }({\text{F}}_{\text{a}}{\text{C}}_{\text{a}}\text{+ }{\text{F}}_{\text{p}}{\text{C}}_{\text{p}}{\text{- F}}_{\text{h}}{\text{C}}_{\text{h}})\;{\text{/C}}_{\text{h}}\\ \;{\text{K}}_{\text{m}},\text{ L/h = }[({\text{C}}_{\text{a}}{\text{- C}}_{\text{m}}){\text{/C}}_{\text{m}}]\;\times {\text{F}}_{\text{m}} \end{array}$$

where *F*_a_, *F*_p_, *F*_h_, and *F*_m_ refer to arterial, portal venous, hepatic venous, and mammary blood flows, respectively (L/h); *C*_a_, *C*_p_, *C*_h_, and *C*_m_ refer to arterial venous, portal venous, hepatic venous, and mammary venous AA concentrations (μmol/L).

Data were subjected to mixed procedure analysis by SAS (version 9.2, SAS Institute, Inc., Cary, NC, USA). The statistic model used was


$$\begin{array}{l} Y_{ijk}\;=\;\mu \;+\;G_{i}\;+\;P_{j}\;+\;T_{K}\;+\;e_{ijk} \end{array}$$

where *Y* was the observed value for the *k*^*n*^ treatment, the *j*^*n*^ period, and *i*^*n*^ goat; μ was the overall mean; *G*_*i*_ was the random effect of goat, *P*_*j*_ was the random effect of the period, *T*_*k*_ was the fixed effect of Lys dose effects, and *e*_*ijk*_ was the random error associated with *Y*_*ijk*_. Both linear and quadratic effects of Lys dose were initially tested. When quadratic effects were nonsignificant, they were removed from the model. The results were expressed as least square means (LSM) with standard error of the mean (SEM). Significance was declared at *p* ≤ 0.05 and tendency was declared at *p* ≤ 0.1. Multiple comparisons between treatment means were made using the Tukey method.

## Results

### Milk Yields and Composition

Removal of Lys from the infused mixture decreased the production of milk, milk protein, milk fat, milk lactose, and the content of milk protein linearly (*p* < 0.05, Table 3). Production of milk, milk protein, milk fat, and milk lactose in the 100 and 60% treatments were greater than in the 30 and 0% treatments. The content of milk protein in the 100 and 60% treatments were higher than the 0% treatment.

**Table 3:** Effects of graded removal of Lys from the amino acid mixture infused into abomasum on lactation performance of lactating goats.

**Item**	**Percentage of Lys**				**SEM**	***p*** **-value**		
	**100%**	**60%**	**30%**	**0%**		**Treatment**	**Linear**	**Quadratic**
Milk, g/9 h	345.5^a^	334.1^a^	264.0^b^	251.2^b^	46.8	0.004	<0.001	0.612
Milk protein, %	4.017^a^	3.845^a^	3.472^ab^	3.205^b^	0.34	0.152	0.013	0.938
g/9 h	13.97^a^	12.83^a^	9.337^b^	8.199^b^	2.18	0.003	<0.001	0.801
Milk fat, %	4.575	4.205	3.948	3.668	0.42	0.411	0.174	0.998
g/9 h	15.71^a^	14.12^a^	10.35^b^	9.412^b^	2.06	<0.001	<0.001	0.884
Milk lactose, %	5.187	5.285	5.267	5.165	0.18	0.909	0.928	0.464
g/9 h	17.79^a^	17.62^a^	13.71^b^	13.03^b^	2.32	0.035	0.007	0.569
Fat/Protein	1.140	1.161	1.188	1.214	0.17	0.991	0.741	0.971

*^a, b^Least square mean within a row with a different superscript indicate a significant difference (p <0.05) between 100, 60, 30, and 0% of Lys*.

### Arterial-Free AA

Lys removal decreased arterial Lys concentration linearly (*p* < 0.05, Table 4). Removing all Lys from the infusate decreased arterial Lys to about one-fifth of the full mixture infusion. Arterial concentrations of all the other measured AA, BCAA, EAA, NEAA, and TAA were unaffected by Lys removal from the infusions.

**Table 4:** Effects of graded removal of Lys from the amino acid mixture infused into abomasum on amino acid concentrations in the plasma of carotid artery in lactating goats.

**Item, μmol/L**	**Percentage of Lys**				**SEM**	***p*** **-value**		
	**100%**	**60%**	**30%**	**0%**		**Treatment**	**Linear**	**Quadratic**
Lys	113.1^a^	73.61^ab^	44.01^b^	23.98^b^	16.5	0.008	<0.001	0.716
Met	36.78	39.47	41.89	40.78	4.23	0.738	0.307	0.628
Val	197.5	186.8	204.4	215.9	23.3	0.844	0.496	0.580
Leu	130.6	122.8	133.4	136.8	17.3	0.942	0.700	0.692
Ile	61.12	60.59	65.65	69.81	11.2	0.795	0.340	0.683
His	33.20	30.52	33.70	27.57	6.80	0.687	0.431	0.685
Phe	78.89	70.95	74.84	76.89	4.58	0.657	0.845	0.264
Thr	60.30	62.45	69.15	77.83	11.3	0.526	0.137	0.626
Trp	27.39	26.56	29.47	32.97	2.52	0.207	0.173	0.241
Arg	84.38	86.74	87.80	104.5	16.2	0.707	0.304	0.559
Gly	707.2	794.6	775.0	786.5	106	0.905	0.551	0.680
Glu	273.7	282.9	287.6	290.8	27.2	0.909	0.432	0.901
Ser	82.88	93.90	91.38	98.79	10.8	0.770	0.323	0.885
Tyr	65.37	65.80	79.02	73.61	11.6	0.573	0.281	0.833
Ala	118.4	124.6	131.7	129.8	13.6	0.805	0.342	0.755
Pro	154.4	176.3	172.0	172.4	23.5	0.829	0.488	0.554
BCAA	389.2	370.2	403.5	422.4	47.7	0.884	0.547	0.637
EAA	823.2	760.5	784.4	807.0	78.2	0.909	0.884	0.496
NEAA	1,402	1,538	1,536	1,552	145	0.835	0.402	0.664
TAA	2,225	2,299	2,321	2,359	207	0.964	0.583	0.946

*^a, b^Least square mean within a row with a different superscript indicate a significant difference (p <0.05) between 100, 60, 30, and 0% of Lys.*

*BCAA, branched-chain AA (Ile, Leu, and Val); EAA, essential AA; NEAA, nonessential AA; TAA, total AA*.

### Arterial Metabolites, Hormones, and Blood Flow

Arterial concentrations of all measured metabolites and hormones were unaffected, apart from glucagon, which increased linearly, and prolactin, which decreased linearly by Lys removal from the infusions (*p* < 0.05, Table 5). Glucagon in the 0% treatment was greater than the 100% treatment and the prolactin in the 100% treatment was greater than the other three treatments. Graded removal of Lys from the infusate linearly increased mammary blood flow (*p* < 0.05, Table 6) than the 0% group which was greater than the 100% group. Portal and splanchnic blood flow were unaffected by Lys removal from the infusions.

**Table 5:** Effects of graded removal of Lys from the AA mixture infused into abomasum on concentrations of metabolites and hormones in the plasma of carotid artery in lactating goats.

**Item**	**Percentage of Lys**				**SEM**	***p*** **-value**		
	**100%**	**60%**	**30%**	**0%**		**Treatment**	**Linear**	**Quadratic**
Urea N, mmol/L	7.257	7.962	7.390	7.610	0.99	0.851	0.808	0.652
Glucose, mmol/L	4.492	4.490	4.545	4.482	0.18	0.994	0.970	0.885
Total Protein, g/L	74.37	75.27	75.55	78.37	2.64	0.642	0.224	0.627
Albumin, g/L	18.90	19.67	19.15	20.72	1.38	0.543	0.230	0.661
Insulin, μIU/ml	43.91	34.99	44.81	44.98	5.59	0.566	0.755	0.412
Glucagon, pg/ml	169.3^b^	188.3^ab^	199.5^ab^	218.8^a^	12.0	0.076	0.006	0.835
Prolactin, ng/ml	3.437^a^	2.872^b^	3.012^b^	2.763^b^	0.16	0.020	0.009	0.307

*^a, b^Least square mean within a row with a different superscript indicate a significant difference (p <0.05) between 100, 60, 30, and 0% of Lys*.

**Table 6:** Effects of graded removal of Lys from the AA mixture infused into abomasum on splanchnic blood flows in lactating goats.

**Blood flow, L/h**	**Percentage of Lys**				**SEM**	***p*** **-value**		
	**100%**	**60%**	**30%**	**0%**		**Treatment**	**Linear**	**Quadratic**
Portal	98.35	93.58	91.76	99.23	8.35	0.401	0.960	0.105
Splanchnic	120.4	122.3	120.5	127.5	6.40	0.508	0.262	0.482
Mammary	17.03^b^	20.39^ab^	22.29^ab^	24.96^a^	2.13	0.043	0.011	0.990

*^a, b^Least square mean within a row with a different superscript indicate a significant difference (p <0.05) between 100, 60, 30, and 0% of Lys*.

### Net Fluxes of Individual AA Across Tissues

The linear decrease in the Lys infusion rate was accompanied by a linear increase in the Glu infusion rate because the missing Lys was replaced by an equal mole of Glu. Net fluxes of individual AA across the PDV, heptic tissues (HEP), TSP, MG, and in milk are presented in Table 7. Graded removal of Lys from the infusate linearly decreased the net fluxes of PDV, HEP, TSP, MG, and milk (*p* < 0.05). Removing all Lys from the infusate decreased the net fluxes of PDV and TSP to about one-sixth of the full mixture infusion. Upon removal of the liver, the amounts of Lys decreased about 85% from 100 to 0% treatment. Mammary uptake of Lys decreased sharply when the infusion of Lys decreased from 60 to 30%. The hepatic removal of Arg was linearly affected by the graded removal of Lys (*p* < 0.05). Net fluxes across the TSP of Leu tended to decrease linearly with the graded removal of Lys (0.05 < *p* < 0.1). Across the MG, the graded removal of Lys caused a linear increase in the uptake of Ile and Val (*p* < 0.05). All the other net fluxes of AA were unaffected by Lys removal from the infusions.

**Table 7:** Effects of graded removal of Lys from the AA mixture infused into abomasum on splanchnic and mammary net fluxes of amino acids in lactating goats.

**Item**, **μmol/h**		**Percentage of Lys**				**SEM**	***p*** **-value**		
		**100%**	**60%**	**30%**	**0%**		**Treatment**	**Linear**	**Quadratic**
Lys	Rate^1^	2,077^a^	1,225^b^	593.5^c^	0^d^	150	<0.001	<0.001	0.822
	PDV^1^	1,523^a^	1,090^b^	691.4^c^	252.0^d^	120	<0.001	<0.001	0.475
	HEP^1^	−152.5^d^	−103.4^c^	−69.41^b^	−21.66^a^	11.0	<0.001	<0.001	0.264
	TSP^1^	1,369^a^	986.5^b^	622.2^c^	231.8^d^	119	<0.001	<0.001	0.602
	MG^1^	−759.6^c^	−705.2^c^	−492.5^b^	−335.0^a^	91.3	0.019	0.001	0.520
	Milk^1^	552.5^a^	537.0^a^	398.7^b^	310.6^b^	45.5	0.002	<0.001	0.442
Ile	Rate	1,442	1,400	1,354	1,420	168	0.984	0.865	0.761
	PDV	1,023	1,081	966.1	858.6	128	0.656	0.306	0.452
	HEP	−110.9	−65.85	−58.65	−71.15	55.6	0.910	0.570	0.622
	TSP	910.6	1,015	907.4	792.0	122	0.623	0.436	0.325
	MG	−493.9^a^	−533.1^ab^	−576.6^ab^	−664.2^b^	53.9	0.079	0.044	0.702
	Milk	305.9	294.7	293.6	325.6	54.9	0.973	0.824	0.685
Val	Rate	2,186	2,123	2,053	2,154	244	0.984	0.865	0.761
	PDV	1,307	1,446	1,344	1,345	153	0.926	0.946	0.621
	HEP	176.9	162.6	126.2	216.6	55.4	0.423	0.887	0.104
	TSP	1,482	1,608	1,470	1,573	200	0.937	0.965	0.853
	MG	−814.6^a^	−1,002^ab^	−1,058^ab^	−1,182^b^	122	0.073	0.067	0.563
	Milk	485.8	445.9	509.2	414.9	101	0.960	0.746	0.776
Met	Rate	775.5	752.9	728.1	764.2	90.5	0.984	0.865	0.761
	PDV	526.3	555.5	516.5	478.0	56.0	0.807	0.482	0.496
	HEP	−242.3	−255.3	−238.8	−202.5	28.9	0.621	0.321	0.352
	TSP	283.7	300.2	277.7	277.3	40.0	0.974	0.826	0.796
	MG	−241.5	−265.4	−254.6	−240.1	33.7	0.943	0.954	0.549
	Milk	215.0	237.2	223.0	208.9	34.4	0.943	0.861	0.572
Leu	Rate	2,780	2,699	2,610	2,740	311	0.984	0.865	0.761
	PDV	1,937	1,737	1,521	1,567	192	0.447	0.119	0.615
	HEP	−118.9	−160.5	−155.2	−163.4	21.3	0.793	0.376	0.649
	TSP	1,816	1,572	1,366	1,413	194	0.389	0.092	0.538
	MG	−1,024	−1,108	−972.3	−1,001	160	0.919	0.882	0.928
	Milk	667.2	648.5	544.5	478.0	117	0.863	0.460	0.892
His	Rate	745.5	723.8	699.9	734.7	83.3	0.984	0.865	0.761
	PDV	576.9	587.0	531.6	568.6	63.8	0.932	0.771	0.887
	HEP	−269.1	−224.9	−251.0	−225.8	33.3	0.748	0.450	0.761
	TSP	307.5	362.1	280.6	345.3	51.8	0.687	0.867	0.999
	MG	−231.2	−305.4	−238.8	−280.7	50.7	0.702	0.676	0.706
	Milk	205.6	273.6	204.7	243.4	47.9	0.703	0.792	0.705
Phe	Rate	2,290	2,223	2,150	2,257	256	0.984	0.865	0.761
	PDV	1,801	1,696	1,634	1,696	198	0.946	0.638	0.701
	HEP	−1,086	−830.7	−995.1	−928.9	149	0.674	0.594	0.503
	TSP	713.8	865.7	639.4	872.3	159	0.782	0.971	0.879
	MG	−626.9	−712.2	−603.0	−607.7	93.6	0.982	0.708	0.844
	Milk	650.0	631.6	595.0	510.1	69.6	0.889	0.415	0.911
Thr	Rate	1,330	1,291	1,249	1,310	148	0.984	0.865	0.761
	PDV	837.6	832.2	782.3	731.6	75.6	0.735	0.274	0.695
	HEP	−330.6	−334.0	−295.7	−280.5	49.1	0.834	0.386	0.781
	TSP	506.2	498.2	486.7	454.0	42.6	0.831	0.367	0.728
	MG	−461.4	−459.6	−413.7	−403.9	46.9	0.845	0.438	0.973
	Milk	416.7	405.0	329.2	304.1	59.2	0.444	0.310	0.855
Trp	Rate	299.5	290.8	281.2	295.2	33.5	0.984	0.865	0.761
	PDV	179.1	159.9	187.2	155.6	20.6	0.661	0.628	0.824
	HEP	−64.70	−34.50	−74.97	−76.01	27.9	0.702	0.594	0.507
	TSP	114.4	125.4	112.2	80.19	32.2	0.781	0.439	0.465
	MG	−116.6	−51.98	−84.32	−68.19	21.9	0.246	0.244	0.286
	Milk	90.92	59.73	76.82	60.69	15.9	0.436	0.551	0.367
Arg	Rate	840.8	846.4	789.5	828.6	93.9	0.984	0.865	0.761
	PDV	602.9	609.6	556.0	515.0	83.0	0.836	0.390	0.712
	HEP	−221.5	−173.0	−159.9	−138.2	26.9	0.201	0.029	0.635
	TSP	381.0	436.6	402.1	379.6	72.6	0.938	0.939	0.564
	MG	−319.6	−489.8	−463.2	−463.9	76.0	0.412	0.211	0.286
	Milk	183.0	166.7	159.5	196.4	56.2	0.691	0.496	0.352
Gly	Rate	972.5	944.2	913.1	958.4	109	0.984	0.865	0.761
	PDV	658.0	624.2	649.3	629.8	93.3	0.318	0.857	0.210
	HEP	−913.1	−894.7	−857.4	−902.8	124	0.894	0.621	0.874
	TSP	−255.1	−270.5	−208.1	−273.0	71.1	0.923	0.909	0.697
	MG	−293.8	−240.5	−283.0	−308.5	135	0.424	0.713	0.247
	Milk	275.9	219.9	262.7	218.9	71.4	0.905	0.978	0.631
Glu	Rate	1,381^b^	1,746^ab^	1,983^ab^	2,390^a^	211	0.049	0.004	0.767
	PDV	408.0	371.6	636.7	319.2	136	0.402	0.982	0.408
	HEP	813.1	701.6	913.1	953.8	177	0.751	0.442	0.590
	TSP	1,219	1,073	1,550	1,283	172	0.308	0.438	0.905
	MG	−839.6	−897.8	−892.3	−826.1	185	0.989	0.973	0.730
	Milk	1,292	1,150	1,116	892.3	241	0.838	0.421	0.854
Ser	Rate	1,971	1,914	1,851	1,943	220	0.984	0.865	0.761
	PDV	1,313	1,489	1,472	1,339	180	0.859	0.888	0.389
	HEP	−822.8	−929.1	−1,009	−826.3	131	0.709	0.827	0.298
	TSP	489.2	559.8	463.8	515.7	105	0.924	0.991	0.884
	MG	−363.2	−354.6	−318.6	−223.0	67.2	0.464	0.142	0.456
	Milk	501.2	445.8	401.4	395.4	93.5	0.843	0.360	0.841
Tyr	Rate	0	0	0	0	–	–	–	–
	PDV	718.8	540.8	531.8	766.8	115	0.389	0.891	0.087
	HEP	−397.6	−230.0	−240.8	−398.7	71.0	0.209	0.921	0.033
	TSP	320.8	310.9	291.0	370.5	63.2	0.835	0.666	0.482
	MG	−172.8	−168.0	−151.1	−139.7	31.1	0.866	0.391	0.858
	Milk	164.2	162.4	152.0	150.0	33.6	0.986	0.713	0.969
Pro	Rate	2,966	2,880	2,785	2,923	331	0.984	0.865	0.761
	PDV	2,025	1,747	1,704	1,706	180	0.550	0.191	0.476
	HEP	−743.5	−605.9	−515.8	−615.7	127	0.629	0.369	0.394
	TSP	1,279	1,141	1,188	1,097	176	0.897	0.495	0.899
	MG	−586.3	−549.4	−763.0	−546.1	135	0.639	0.870	0.595
	Milk	809.7	767.1	655.6	570.7	139	0.605	0.475	0.851
Ala	Rate	1,299	1,261	1,219	1,279	145	0.984	0.865	0.761
	PDV	1,245	1,433	1,215	1,172	213	0.642	0.792	0.297
	HEP	−519.8	−736.9	−566.7	−352.0	169	0.484	0.445	0.181
	TSP	724.1	696.3	648.8	827.1	141	0.522	0.164	0.956
	MG	−507.9	−504.2	−513.1	−609.7	73.9	0.707	0.354	0.467
	Milk	595.5^a^	366.6^b^	277.3^b^	327.6^b^	61.6	0.015	0.278	0.620

*^a–d^Least square mean within a row with a different superscript indicate a significant difference (p <0.05) between 100, 60, 30, and 0% of Lys.*

*PDV, portal-drained-viscera; HEP, hepatic tissues; TSP, splanchnic tissues; MG, mammary gland; Milk, milk output*.

### PDV, Liver, and Mammary Clearance Rates of AA

The PDV clearance rate of Lys and Val decreased linearly with the graded removal of Lys (*p* < 0.05, Table 8). Numerically, the PDV clearance rate of Lys decreased by 2.43-folds for the 30% Lys treatment compared with that of complete mixture infusion. All the other measured EAA were unaffected with graded removal of Lys.

**Table 8:** Effects of graded removal of Lys from the AA mixture infused into abomasum on PDV, liver and mammary amino acids clearance rates of lactating goats.

**Item, L/h**		**Percentage of Lys**				**SEM**	***p*** **-value**		
		**100%**	**60%**	**30%**	**0%**		**Treatment**	**Linear**	**Quadratic**
PDV	Lys	7.219^a^	5.115^ab^	2.977^b^	–	1.22	0.085	0.015	0.770
	Met	5.838	4.700	5.264	5.698	1.10	0.365	0.332	0.183
	Val	4.226^a^	3.387^ab^	3.250^ab^	2.567^b^	0.51	0.096	0.009	0.956
	Leu	5.669	6.826	7.291	7.785	1.24	0.493	0.113	0.803
	Ile	5.838	4.700	5.264	5.698	1.09	0.845	0.972	0.404
	His	4.716	3.904	5.029	3.398	1.27	0.327	0.373	0.627
	Phe	5.137	6.007	5.409	3.871	1.11	0.540	0.392	0.220
	Thr	7.096	6.330	6.537	5.506	1.10	0.693	0.258	0.863
	Trp	4.045	4.591	2.947	3.416	0.56	0.134	0.180	0.754
	Arg	3.263	2.381	3.342	2.276	1.03	0.784	0.612	0.961
Liver	Lys	1.403	1.399	1.381	0.949	0.24	0.294	0.239	0.237
	Met	6.271	6.032	5.448	5.004	0.78	0.632	0.178	0.813
	Val	−0.848	−0.758	−0.813	−0.998	0.27	0.830	0.566	0.438
	Leu	0.854	0.565	0.504	0.656	0.54	0.904	0.626	0.547
	Ile	1.555	0.731	0.907	0.834	0.80	0.809	0.449	0.642
	His	7.534	6.683	8.216	8.034	1.08	0.616	0.557	0.610
	Phe	13.12	10.97	12.37	11.69	2.35	0.884	0.699	0.688
	Thr	5.482	5.385	4.350	3.542	1.04	0.524	0.146	0.637
	Trp	2.318	1.477	2.581	2.284	0.98	0.839	0.832	0.701
	Arg	3.181	2.337	1.932	1.275	0.81	0.112	0.008	0.979
Mammary	Lys	16.03^b^	17.23^b^	21.61^ab^	31.73^a^	6.79	0.068	0.014	0.208
	Met	11.89	11.67	8.877	8.291	2.58	0.639	0.215	0.840
	Val	5.877	7.741	7.213	6.755	1.20	0.142	0.185	0.244
	Leu	16.64	19.18	14.67	15.56	4.62	0.806	0.626	0.731
	Ile	17.64	23.96	20.91	19.08	5.73	0.748	0.878	0.323
	His	14.64	20.19	12.21	20.14	5.85	0.485	0.652	0.874
	Phe	3.598^ab^	3.975^a^	3.251^ab^	3.051^b^	0.98	0.075	0.084	0.172
	Thr	15.37^a^	12.34^ab^	9.656^ab^	7.135^b^	1.83	0.031	0.002	0.809
	Trp	5.784	2.246	3.420	2.389	1.19	0.193	0.106	0.308
	Arg	6.393	8.317	8.404	5.397	1.61	0.332	0.734	0.074

*^a, b^Least square mean within a row with a different superscript indicate a significant difference (p <0.05) between 100, 60, 30, and 0% of Lys*.

The liver clearance rate of Arg decreased linearly (*p* < 0.05). Graded removal of Lys from the infusate had no significant effect on liver clearance rates of the other EAA measured.

Graded removal of Lys from the infusate increased the mammary clearance rate of Lys linearly (*p* < 0.05). Numerically, the mammary clearance rate of Lys increased by 2.06-folds for the 0% Lys treatment compared with that of complete mixture infusion. The mammary clearance rate of Thr decreased linearly (*p* < 0.05) while mammary clearance for Phe tended to decrease linearly (0.05 < *p* < 0.1) with decreasing the infusion of Lys.

### U:O of AA

Graded removal of Lys from the infusate decreased linearly mammary U:O of Lys to a significant level (*p* < 0.05; Table 9). The mammary U:O of Lys in the 100% treatment was greater than 30% and 0% treatments. Graded removal of Lys from the infusate linearly increased the mammary U:O of Leu and Ile. Treatments affected the U:O of Arg quadratically (*p* < 0.05). U:O increased and then decreased with decreasing Lys content in the infusate. Treatments had no significant effects on mammary U:O of the other AA measured.

**Table 9:** Effects of graded removal of Lys from the AA mixture infused into abomasum on the ratio of mammary uptake to milk output in lactating goats.

**Item**	**Percentage of Lys**				**SEM**	***p*** **-value**		
	**100%**	**60%**	**30%**	**0%**		**Treatment**	**Linear**	**Quadratic**
Lys	1.455^a^	1.314^ab^	1.246^b^	1.094^b^	0.09	0.042	0.027	0.538
Met	1.150	1.125	1.187	1.135	0.07	0.936	0.964	0.894
Val	1.914	2.368	2.341	2.300	0.32	0.740	0.592	0.322
Leu	1.684^b^	1.724^b^	1.959^ab^	2.091^a^	0.21	0.058	0.047	0.885
Ile	1.605	1.875	2.099	2.170	0.38	0.047	0.025	0.508
His	1.159	1.114	1.160	1.218	0.07	0.783	0.513	0.424
Phe	0.909	0.906	0.896	1.008	0.06	0.604	0.355	0.368
Thr	1.145	1.162	1.328	1.382	0.12	0.467	0.122	0.761
Arg	1.867^b^	2.970^a^	2.993^a^	2.513^ab^	0.25	0.023	0.126	0.009
Gly	1.219	1.404	1.696	1.817	0.66	0.458	0.811	0.513
Glu	0.746	0.756	0.799	0.927	0.15	0.701	0.351	0.489
Ser	0.832	0.838	0.774	0.740	0.19	0.981	0.692	0.888
Tyr	1.058	1.060	1.080	0.885	0.10	0.511	0.299	0.322
Ala	1.281	1.367	2.260	1.869	0.41	0.343	0.166	0.719
Pro	0.729	0.675	1.164	0.992	0.18	0.280	0.151	0.994

*^a, b^Least square mean within a row with a different superscript indicate a significant difference (p <0.05) between 100, 60, 30, and 0% of Lys*.

## Discussion

### Milk Protein Production

As indicated in previous studies, the extent of Lys deficiency in the experiment was limited by the Lys supply from the basal diet, and there was little chance of having a significant change in milk protein production. For example, of the seven experiments that investigated the response of milk protein to varied post-ruminal Lys supply since 2000, six experiments observed no significant effects (16, 33–37), and the other reported lower milk protein production for the low MP diet supplemented with Lys compared with that of the MP-adequate diet (38). Therefore, such experimental procedures are inappropriate if we only want to investigate the effects of individual EAA on milk protein synthesis. To avoid the limitation of basal diets on the extent of individual AA deficiency, one experiment was designed by subtracting approximately 45% of each EAA from the full mixture of EAA infused into the cows. This experiment was to investigate the effect of the post-ruminal supply of individual EAA on milk protein production and found that the removal of Lys caused the greatest depression in milk protein production (39). Another experiment found that graded removal of Lys from an intravenously infused amino acid mixture linearly decreased milk protein production (3). In this study, graded removal of Lys from the infusate also linearly decreased milk protein production. Considering the concomitant linear decrease in mammary Lys uptake (Table 7), it seemed reasonable to interpret the result as a mass action of Lys supply. Meanwhile, we observed a linear increase in circulating glucagon and a decrease in circulating prolactin by a graded removal of Lys from the infusate in the present study. It has been observed that the infusion of glucagon decreased milk protein production in lactating cows (40, 41). Prolactin also plays an important role in mammary development and lactation (42). Inhibition of the release of prolactin significantly reduced milk protein yield in dairy goats and cows (43–46). Thus, Lys supply may exert a portion of its effects on milk protein synthesis through glucagon and prolactin. As mentioned above, a variation in post-ruminal Lys supply can affect milk protein synthesis through mass action with Lys and a change in hormonal status.

### Splanchnic and Mammary Net Fluxes of Amino Acids

As revealed by previous studies, decreasing the post-ruminal supply of an EAA would generally decrease the circulating concentration of that specific EAA (3, 36, 47). Consistent with these expectations, arterial concentrations of Lys decreased linearly with the removal of Lys in the present study. Compared with the complete mixture of the 100% treatment, the removal of all Lys reduced arterial Lys concentrations to 21%. Arterial concentrations of other EAAs were not affected by treatment, indicating that the metabolism of individual EAA usually had an independent effect on protein synthesis (48).

The splanchnic and mammary net flux of AA is closely related to blood flow. The contribution of portal blood flow to splanchnic blood flow ranges from 75 to 85% (49–51). In this study, portal blood flow (95.7 L/h) was 78% of splanchnic blood flow (122.7 L/h), which was consistent with previous studies of lactating ruminants. The splanchnic blood flow regulated by energy supply rather than protein supply (52) had no response to graded removal of Lys. Graded removal of Lys linearly increased mammary blood flow. This phenomenon was observed for Met removal (24), Lys removal (3), His deficiency (53), and Leu deficiency (27). Therefore, increased mammary blood flow appears to be a common response in response to deficiencies of individual EAA (54), which helps to mitigate the deficiency. NO concentration in the mammary vein plasma was linearly elevated by Met removal (24) and Lys removal (3) in the previous experiments. This may at least partially explain the elevated mammary blood flow as NO has been identified as one of the local vasodilatory compounds (55). The mechanisms of AA that induced the increased MG blood flow need further investigation.

Amino acid metabolism by the PDV tissue affects the net supply and composition of AA entering the bloodstream (4). Gut use of AA also varies by different AAs with the loss of His being very small, while the loss of Leu, Thr, and some NEAA was much greater (56). The same phenomenon occurred in this experiment, where the net recovery ratio of His was greatest at about 80%, but the BCAA only averaged about 63%. If the PDV flux of the 0% Lys treatment was taken as the basal absorption, the metabolic loss of Lys in the PDV is 39.2, 31.6, and 26.3% for 100, 60, and 30% treatments, respectively. Meanwhile, the PDV clearance rates for Lys decreased linearly from 7.219 to 2.977 L/h. Thus, decreases in post-ruminal Lys supply resulted in decreased utilization by the digestive tract and increased relative availability of Lys for other tissues due to decreased affinity of Lys in PDV. PDV flux of other AAs was not significantly affected, indicating that the metabolic loss rate of PDV can be altered by adjusting an EAA supply.

If all AAs are considered together, then the liver removes an average of about 45% of portal absorption (4). However, this value hides the considerable variation in how the individual AA is handled and the impact this has on post-hepatic supply and metabolism. In this experiment, the net removal of Met, His, Phe, Thr, Trp, and Arg by the liver was relatively large, between 35 and 60%, while the removal of Lys and BCAA by the liver was very small. Although the net liver flux of Lys decreased linearly, the removal ratio of portal absorption changed small, which was about 10% at the four treatments. At the same time, the clearance rate of Lys by the liver also showed a nonsignificant change with decreasing Lys supply (Table 8). This suggests that the liver can remove Lys in a fixed proportion in lactating ruminants.

Post-liver supply was greater than mammary uptake, which exceeded milk output, as is usually observed for Lys (4). The gradual removal of Lys resulted in a linear decrease in the amount of Lys delivered to peripheral tissue (net TSP flux) and mammary uptake (Table 8) in this experiment, which was similar to a previous study (34). The mammary uptake amount of Lys accounts for more and more peripheral tissue supply with decreasing post-ruminal Lys supply, indicating that the utilization of Lys in peripheral tissues (non-mammary) is reduced. Mammary uptake and milk output of Lys was even higher than the post-liver supply in the 0% treatment, this may be due to the mobilization of body protein reserves to meet the requirement of lactation (51). There were no significant changes in the mammary uptake of Lys between 100 and 60% treatments despite large declines in arterial Lys concentrations of about 35% between 100 and 60% treatments. Changes in mammary blood flow and mammary affinity compensated for part of the varied supply of individual EAA, thereby mitigating the responses (3, 53). However, there was a limit to this buffering because the mammary uptake of Lys decreased significantly when the post-ruminal supply of Lys was reduced to 30%.

Branched-chain amino acids and Lys are taken up in excess by the MGs (57). It is known that Lys can be used to support necessary NEAA synthesis in MGs (17) and its utilization in mammary is sensitive to nutrient supply (58). The mammary U:O of Lys decreased linearly from 1.45 to 1.09, which is similar to a previous study that U:O decreased when decreasing doses of Lys supply (34, 57, 59). Although MGs could decrease U:O, the values always exceeded unity, even at 0% treatment. This suggested that there are other roles for Lys within the MG, such as supporting necessary NEAA synthesis (17). Meanwhile, increased mammary uptake of Ile and Val and increased U:O of Leu and Ile compensated for the lowered metabolism of Lys because BCAA could provide N for NEAA synthesis (34).

### Mechanisms to Mitigate the Lys Deficiency

**Figure 2:**
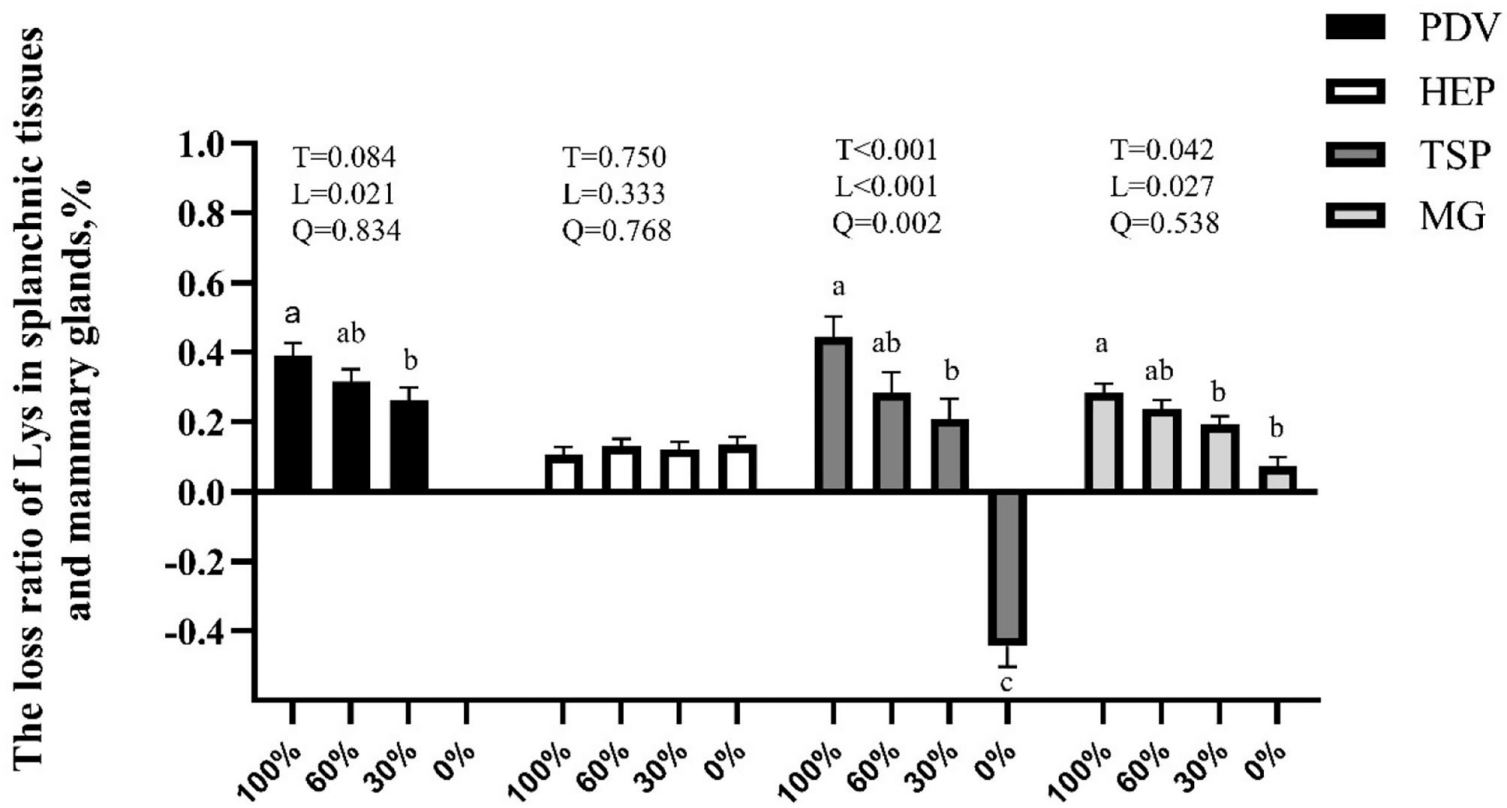
Effects of graded removal of Lys from the amino acid (AA) mixture infused into abomasum on the loss ratio of Lys in splanchnic tissues and mammary glands of lactating goats. PDV, portal-drained-viscera; HEP, hepatic tissues; TSP, splanchnic tissues; MG, mammary gland. ^ac^Least square mean within a row with a different superscript indicate a significant difference (*p* < 0.05) between 100, 60, 30, and 0% of Lys.

The main innovation of this study was that we can observe the route of AA absorbed into milk protein and the response of amino acid metabolism in the PDV, liver, and MG to the change of Lys supply by using the multi-catheterized model of dairy goats, this provides a new strategy to improve postabsorptive N efficiency. The infusion rate of Lys decreased by about 2,100 μmol/h from 100 to 0% treatment. Nonetheless, mammary uptake only decreased by 424 μmol/h while Lys in milk protein secretion decreased only by about 242 μmol/h from 100 to 0% treatment. According to the outcomes of this experiment, there are several pathways to mitigate Lys deficiency in lactating ruminants. First, lactating ruminants adapted to Lys deficiency by reducing net utilization or catabolism in PDV and peripheral tissues (except mammary, Figure 2). There may be a limit to this adaptation; however, as mammary uptake was still impacted negatively when the post-ruminal supply of Lys decreased to 30% and 0%. Second, lactating ruminants enhanced mammary uptake ability to Lys due to increased mammary blood flow and mammary affinity (Tables 6, 8). Lastly, the utilization or catabolism of Lys in the MG is reduced along with the increased mammary uptake and U:O of BCAA to compensate for the lowered metabolism of Lys. From all of the above, we can include that lactating ruminants have a priority to meet the requirement of lactation.

## Conclusion

Lactating ruminants use several mechanisms to mitigate Lys deficiencies, including reduced utilization of Lys by PDV and peripheral tissues (including MGs), linear increases in mammary blood flow, and mammary affinity for Lys. This can include the lactating ruminants having a priority to meet the requirement of lactation. Graded removal of Lys from the abomasum-infused AA mixture linearly decreased milk protein production. The decrease was a combinative result of mass action with Lys and changed hormonal status with increased glucagon and decreased prolactin. Further work is required to delineate external hormonal effects and intracellular cell signaling effects.

## Data Availability Statement

The original contributions presented in the study are included in the article/supplementary material, further inquiries can be directed to the corresponding author.

## Ethics Statement

The animal study was reviewed and approved by Institutional Animal Care and Use Committee of Shandong Agricultural University.

## Author Contributions

YL and ZW: conceptualization and methodology. YL and CL: formal analysis. YL: writing the original draft preparation. XL, ZH, QH, and ZW: writing—review and editing. XL and ZW: project administration and supervision. All authors contributed to the article and approved the submitted version.

## Funding

This work was funded by the National Natural Science Foundation of China under Project No. 31772623 and by the China Agriculture Research System of MOF and MARA.

## Conflict of Interest

The authors declare that the research was conducted in the absence of any commercial or financial relationships that could be construed as a potential conflict of interest.

## Publisher's Note

All claims expressed in this article are solely those of the authors and do not necessarily represent those of their affiliated organizations, or those of the publisher, the editors and the reviewers. Any product that may be evaluated in this article, or claim that may be made by its manufacturer, is not guaranteed or endorsed by the publisher.
